# Another Death from Chloroform

**Published:** 1861-02

**Authors:** 


Another Death from Chloroform.—Dr. Fano reports the history of a patient in the *Gazette Hebdomadaire*, of November 30, who died from chloroform. The patient had been suffering from incurvature of the toe-nail, and Dr. Fano put him under chloroform for the purpose of relieving the incurvation of the nail by that very painful operation, originated by Dupuytren. Everything was favorable for the inhalation: the patient was in the horizontal position, on a bed opposite a large open window. The charpie wet with chloroform did not obstruct the mouth, being applied only to the nose. Dr. Lombard assisted in watching the pulse. The patient soon became insensible, when Dr. Fano proceeded with the operation. When he had torn out the nail the patient gave a groan, when Dr. F., looking at his face, found it pale, his pulse at the wrist extinguished, and no pulsation of the heart. Various means were used to resuscitate the patient, as dashing cold water in his face, washing his face with vinegar and water; artificial respiration was also practiced. After the lapse of some moments the patient made three or four respirations, without any return of the pulse, or any intelligence. All means were used, but without success. After some minutes the lips became violet, but this color was not present at the moment when attention was called to him by his groaning. "The autopsy was made by Dr. Tardieu, *who found old adhesions between the lungs and thoracic walls to a great extent, and a pulmonary apoplexy.*"

The editors of the *Gazette Hebdomadaire*, from which journal we have taken the main facts of this case, make the following remarks on it: "It appears evident to every one after reading this history, that our confrères took all the necessary precautions to ensure safety in the inhalation of the anæsthetic: open windows, horizontal position, the chloroform administered in small successive quantities, incessant watching of the pulse, etc. Still further, when the dangerous results manifested themselves, they did what science and experience counsel, to avert the catastrophe. Their conscience may then rest calm in the face of an event so terrible."

Is it not about time that chloroform should be laid aside? Can any one pretend to lay down rules for its safe administration?—*Cincinnati Lancet and Observer.*

